# Randomised, multicentre, placebo-controlled trial of fenofibrate for treatment of diabetic macular oedema with economic evaluation (FORTE study): study protocol for a randomised control trial

**DOI:** 10.1136/bmjopen-2024-089518

**Published:** 2024-12-20

**Authors:** Helen Nguyen, Richard Kha, Bamini Gopinath, Paul Mitchell, Gerald Liew

**Affiliations:** 1 Westmead Institute for Medical Research, Westmead, New South Wales, Australia; 2 The George Institute, Sydney, New South Wales, Australia

**Keywords:** DIABETES & ENDOCRINOLOGY, OPHTHALMOLOGY, Diabetic retinopathy, Diabetes Mellitus, Type 2, Medical retina

## Abstract

**Introduction:**

Diabetic macular oedema (DMO), a serious ocular complication of diabetic retinopathy (DR), is a leading cause of vision impairment worldwide. If left untreated or inadequately treated, DMO can lead to irreversible vision loss and blindness. Intravitreal injections using antivascular endothelial growth factor (anti-VEGF) and laser are the current standard of treatment for DMO. These treatments are costly and invasive and must be repeated over several years with a high service load. Fenofibrate has been shown to reduce the progression of DR. However, there is a lack of high-quality data on the effects of fenofibrate on established DMO. This study aims to evaluate the effectiveness of oral fenofibrate for the treatment of DMO.

**Methods and analysis:**

This randomised double-blind, placebo-controlled trial recruited 204 patients with DMO across three different clinics in Sydney. Participants will be randomly allocated in a 1:1 ratio to intervention and control groups. The intervention group will receive oral fenofibrate (145 mg) taken once daily for 24 months, while the control group will receive placebo tablets taken once daily for 24 months. Standard care with anti-VEGF injections, focal lasers or observation will also be provided to all participants regardless of their group allocation. The primary outcome is the reduction in DMO measured using central macular subfield thickness (CSMT) on optical coherence tomography imaging at 24 months. Secondary outcomes at 24 months include the proportion of eyes with CSMT <250 µm, number of anti-VEGF injections, number of laser sessions needed, best-corrected visual acuity letter score gains, rates of adverse events, progression in DR lesions and changes in quality of life measures. Comparison between groups will be evaluated using analysis of variance. Multiple regression analyses adjusting for age, glycated haemoglobin, number of injections and other covariates will also be performed.

**Ethics and dissemination:**

Ethics approval has been granted by the University of Sydney Human Ethics Committee (HREC-2019/892), and the trial has been registered with the Australia New Zealand Clinical Trials Registry (ACTRN12618000592246). The study adheres to the principles of the Helsinki Declaration and the National Health and Medical Research Council National Statement on Ethical Conduct in Human Research. Trial results will be disseminated to the public in de-identified form through publications in peer-reviewed journals.

**Trial registry name:**

Australian New Zealand Clinical Trials Registry.

**Trial registration number:**

ACTRN12618000592246.

**STRENGTHS AND LIMITATIONS OF THIS STUDY**

- The fenofibrate for the treatment of diabetic macular oedema study is the first adequately powered randomised controlled trial focusing on the major visual endpoint of diabetic macular oedema (DMO).
- The study will use objective measurements for accurate assessment of DMO and stratification by the major confounders of treatment types.
- There is masking of all research personnel, including treating physicians, and measurement of clinical outcomes that are most important to patients, including best-corrected visual acuity, number of injections and quality of life measures.
- The main limitation is that recruitment is limited to participating clinics located mostly in Sydney’s western suburbs. Therefore, it may not be entirely generalisable to other populations with diabetic retinopathy.

## Introduction

 Diabetic macular oedema (DMO), a sight-threatening form of diabetic retinopathy (DR), is the main cause of vision impairment in diabetic eye disease and affects 3%–5% of patients with diabetes (an estimated 60 000 patients in Australia and 20 million worldwide).^1 2^ Preclinical DMO starts as a small amount of oedema fluid with mild impact on vision and progresses over months to become the more severe clinical DMO with marked oedema swelling and severe vision loss. If left untreated or treated insufficiently, damage to vision can become irreversible and may lead to legal blindness.^3^ Many patients with DMO are young to middle-aged, have poor vision and are at risk of losing the ability to drive or work, resulting in further distress and financial hardship.^3^ The total annual direct financial cost of diabetes in Australia is estimated to be over AUD$14.6 billion, of which a substantial proportion is due to vision impairment from diabetic eye disease.^4^ This sum is even greater when factoring in indirect costs from vision impairment in working-age adults, such as loss of income, long-term carer support and lost economic productivity.^4^

Some patients with mild DMO do not require active treatment, while for those that have more severe forms, the current standard treatment is intravitreal antivascular endothelial growth factor therapy (anti-VEGF) injections and/or focal laser.^5^ Some patients who do not respond to these interventions may respond to second-line treatment with intravitreal steroid injections. All these treatments are costly and invasive with significant risk of causing vision loss and other complications. Treatments must also be repeated over several years with a high service load. The licensed anti-VEGF agents such as ranibizumab, aflibercept and faricimab are expensive, and usage is expected to increase alongside the ageing population and increasing prevalence of diabetes.

Management of DMO could be greatly improved with an agent that acts synergistically with anti-VEGF injections or lasers, thus lowering the number of treatment sessions needed. Fenofibrate is a peroxisome proliferator-activated receptor alpha (PPAR-α) agonist that shows promise for the treatment of DMO. It is an orally delivered fibric acid which has been used to treat dyslipidaemia for decades. It has an excellent safety profile, is well tolerated and is inexpensive.^6^ After absorption, fenofibrate activates PPAR-α, which is a transcription factor responsible for the expression of genes regulating lipid metabolism.^7^ Activation of PPAR-α by fenofibrate upregulates the synthesis of proteins, resulting in reduced low-density lipoproteins and triglyceride levels and increased high-density lipoprotein levels.^7^

The mechanism by which fenofibrate inhibits the progression of diabetic eye disease remains unknown and is thought to be more complex than the serum lipid-lowering effect. Fenofibrate upregulates apolipoprotein A1, which may have a role in reducing the progression of DR by protecting the retina from oxidative stress and lipotoxicity.^8^ Fenofibrate also lowers lipoprotein-associated phospholipase A2, which has been shown to decrease the production of proangiogenic prostaglandins and retinal neovascularisation.^9^ In addition to these lipid-modulating effects, several other non-lipid mechanisms have been proposed to explain the protective effect of fenofibrate in DR. These include decreased endothelial cell permeability, interleukin-1 beta-mediated retinal pigment epithelium (RPE) disruption and nuclear factor kappa B-mediated inflammatory cytokine expression, all of which contribute to a blood-retinal breakdown in DMO.^10 11^ Fenofibrate also downregulates stress-related signalling and apoptosis of endothelial and pericyte cells through adenosine monophosphate-activated protein kinase-dependent pathways to preserve the outer blood-retinal barrier.^10 12^ Moreover, fenofibrate may have a potential role in inhibiting angiogenesis by downregulating VEGF in RPE cells.^13 14^

Two large clinical trials in patients with type 2 diabetes have demonstrated that oral fenofibrate is effective at slowing the progression of DR. The Fenofibrate Intervention and Event Lowering in Diabetes (FIELD) study indicated that fenofibrate reduced the need for the first laser to treat DMO by 31% and reduced the rate of progression of a composite endpoint of DR, DMO and laser treatment by 34%.^15^ The Action to Control Cardiovascular Risk in Diabetes (ACCORD) Eye Study showed that fenofibrate, combined with statin use, reduced the progression of DR lesions by 40%.^16 17^ Both these studies showed fenofibrate has a good safety profile, with the major adverse effect being a transient and reversible elevation of creatinine levels, which was of little clinical significance. Although both trials demonstrate fenofibrate slows the progression of DR, there are some limitations that are worth noting. First, the FIELD study excluded patients with established DMO or severe DR, and neither study used optical coherence tomography (OCT) to assess for DMO, the gold standard procedure in clinical practice. Furthermore, DMO was not a separate endpoint, as the trials examined a composite endpoint of any laser intervention only.

Additionally, a small French randomised clinical trial with a sample size of 110 participants examined the effectiveness of oral fenofibrate in treating and reversing DMO.^18^ This study found suggestive, but non-significant, results that fenofibrate over 12 months reduced the thickness of preclinical DMO by 10% compared with placebo (p=0.59). The study was acknowledged by the authors to be underpowered, and participants receiving anti-VEGF injections or lasers were excluded, limiting the clinical utility. Another study by Srinivasan *et al* in 2018 evaluated the effect of oral fenofibrate on macular thickness and visual function in 50 participants with DMO.^19^ This study found a statistically significant decrease in macular thickness after 6 months but no improvement in visual function.^19^ However, the study was limited by its small sample size and short follow-up duration of 6 months. Therefore, there is no high-quality data on the effects of fenofibrate on established DMO or fenofibrate used in combination with anti-VEGF injections or lasers. This is an important gap to address and determine if fenofibrate can be a reliable treatment option for DMO.

We, therefore, designed a randomised clinical trial, fenofibrate for the treatment of diabetic macular oedema (FORTE), to test the hypothesis of whether the use of oral fenofibrate is effective for treating DMO, with three major prespecified subgroup analyses corresponding to the main clinical indications, that is, in patients receiving observation, laser and anti-VEGF injections. Our study protocol addresses previous limitations by recruiting an adequately powered sample (n>200), having longer follow-up over 24 months, as the effects of fenofibrate take time to develop,^15 16^ and including participants receiving anti-VEGF and laser interventions.

## Materials and methods

### Study design

This phase III study is a multicentre, randomised, placebo-controlled, double-masked, clinical trial with an intervention arm and a control arm, designed to test the efficacy of oral fenofibrate combined with standard treatment (intravitreal anti-VEGF injections, laser and observation) in treating and reversing established DMO. After written consent to participate in the study (see online supplemental file 1), participants will be screened to evaluate patient eligibility, followed by randomisation into the intervention arm or control arm with a 1:1 allocation ratio, using three randomisation strata (anti-VEGF-treated DMO, laser-treated DMO and DMO requiring clinical observation).

#### Inclusion criteria

1. Age ≥25 years at the time of consent.
2. Able to understand and provide written informed consent. Formal interpreters will be organised for participants from non-English-speaking backgrounds.
3. Documented diagnosis of type 2 diabetes.

Inclusion criteria for study eye:

1. Diagnosis of DMO requiring treatment (anti-VEGF or laser) determined by the investigator and with a central macular subfield thickness ≥250 µm.
2. Diagnosis of DMO requiring clinical observation determined by the investigator and with a central macular subfield thickness ≥250 µm but <350 µm.

If both eyes meet all the eligibility criteria, the worse eye (defined as the one with greater central macular subfield thickness or greater area of involvement) as assessed at screening will be selected as the study eye.

#### Exclusion criteria

Patients meeting any of the following criteria will not be eligible for inclusion in this study:

1. Current or prior use of fenofibrate.
2. Pregnancy or intention to become pregnant within the trial period.
3. Contraindication to fenofibrate use. These are very few and include allergic reactions to fenofibrate and a history of severe kidney failure, liver disease and pancreatitis.
4. Other causes of macular oedema include epiretinal membrane, vitreomacular traction, retinal vein occlusion, age-related macular degeneration and cataract surgery in the last 6 months.
5. Previous treatment for DMO in the prior 6 months included focal/grid laser, anti-VEGF injections and intravitreal triamcinolone injections.
6. Active retinal neovascularisation requiring pan-retinal photocoagulation laser therapy in the last 6 months.

### Recruitment

**Figure 1:**
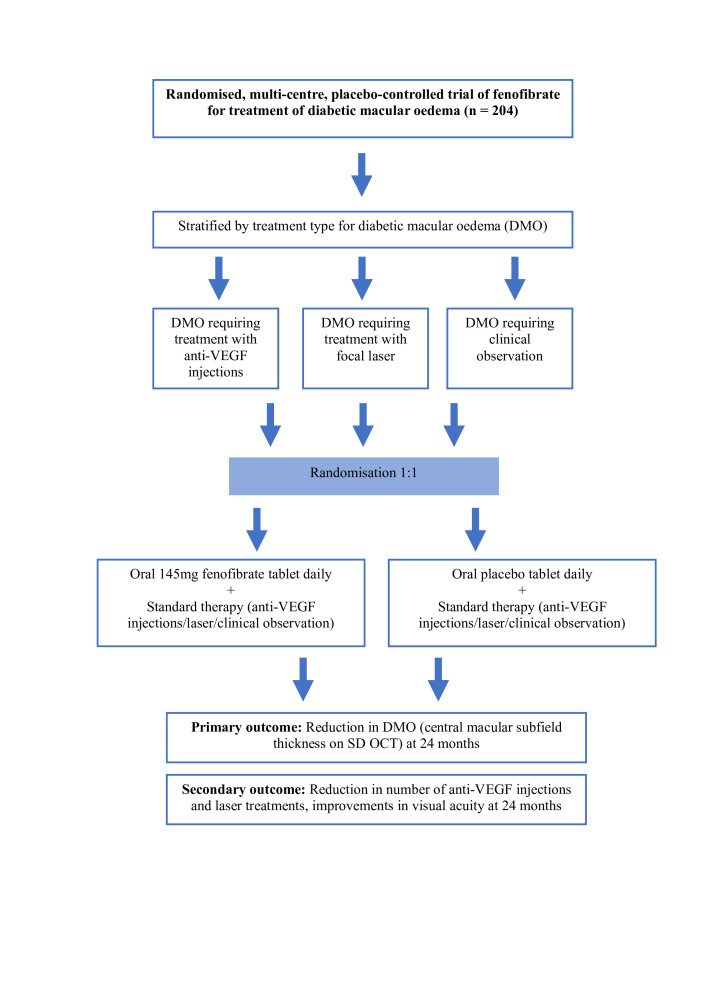
Flow chart for participants in the fenofibrate for the treatment of diabetic macular oedema trial. VEGF, vascular endothelial growth factor; SD-OCT, spectral domain optical coherence tomography.

The flow chart for participants in the FORTE trial is illustrated in figure 1. A total of 204 participants were recruited from the period starting from April 2018 to December 2023. The original study design aimed for recruitment to be completed by December 2021; however, due to the impact of the COVID-19 pandemic and repeated state-wide lockdowns, recruitment was extended by 2 years to reach the target sample size. Follow-up of trial patients will run from the time of recruitment until December 2025 (24 months for all patients). Recruitment and follow-up took place at three ophthalmology clinics located in areas of Sydney with a high prevalence of diabetes.

### Randomisation and masking

Eligible patients who provide written consent will be assigned a participant number that serves as a unique identification number on all study documents. Participants will be randomised and receive their first batch of study medication at their screening/baseline visit.

The randomisation sequence will be generated centrally using permuted blocks of mixed size. Randomisation will occur in three strata reflecting clinical status and standard therapy: anti-VEGF, laser or observation, to ensure equal numbers of drug and placebo allocation within each stratum while maintaining an unpredictable sequence.

A system of tamper-resistant sealed envelopes prepared by the central team will be used to indicate which medication kit number should be dispensed to the next participant recruited within each stratum. The envelopes will be distributed to individual sites as needed and kept in a secured cabinet. When the recruitment process for a participant has been completed and checked, the study coordinator will record core participant and recruitment details before the next envelope, which assigns their medication kit number is opened. The study coordinator and all treating staff will be masked to the actual treatment allocation throughout the study, with only the randomisation and medication supply coordinator unmasked to treatment allocation. Patients can be unmasked if concerns are raised by study personnel and after consultation with the Data Monitoring and Safety Committee, or if hospital admission occurs.

### Intervention and control (placebo) groups

The intervention group will receive oral fenofibrate (145 mg), to be taken once daily for a period of 24 months. These will be dispensed four times during the study period with six bottles containing 30 tablets each dispensed at each relevant visit. Participants will return the empty bottles at key mandated visits to document compliance. Participants randomised to control will receive identically marked and packaged placebo tablets, dispensed in the same bottles and at the same visits. Compliance with tablet taking will also be assessed through pill counting on receipt of empty or partially empty bottles.

Participants in both groups will receive their assigned treatment, that is, observation, anti-VEGF injections or focal laser according to current guidelines.^2^ If participants have centre involving DMO and best-corrected visual acuity (BCVA) worse than 75 Early Treatment Diabetic Retinopathy Study (ETDRS) letters (6/9 Snellen equivalent), participants will receive anti-VEGF injections with aflibercept as this has been shown to be highly effective in previous trials.^20^ Only aflibercept will be used to minimise confounding from use of other anti-VEGF agents.

Treat and extend as recommended by current guidelines will be the protocol followed for this study, with a possible maximum of 24 injections and minimum of three loading doses. Participants with non-centre involving DMO who have clinically significant macular oedema (CSMO) will be allocated to receive focal laser; those who do not have CSMO will be allocated to observation. If participants in either group progress and DMO becomes centre involving, anti-VEGF treatment with aflibercept will be permitted and the number of injections will be documented. Participants will remain in their initially stratified group for the purposes of data analyses.

### Outcome measurements

#### Primary outcomes measured at 24 months

The primary outcome is the reduction in DMO, measured objectively using central macular subfield thickness on spectral domain-OCT (SD-OCT) in patients on fenofibrate compared with those on placebo.

This is an objective measurement in micrometre of the severity of macular oedema and is now a routinely used endpoint in clinical trials.^20 21^ OCT scan protocols will include a dense horizontal linear scan centred on the fovea, and the software will be used to correlate position markers on near infrared to facilitate registration and longitudinal scanning of the same area at each mandated follow-up visit, as in the baseline OCT scan. The readings are highly reproducible, and change of >10 µm is considered to represent ‘real’ change outside the range of measurement error.^22^ At every key visit, masked investigators will record this number on clinical research forms, and the OCT raw data will be sent to the reading centre at the Centre for Vision Research, Westmead Institute for Medical Research (WIMR), University of Sydney, for cross-checking by masked graders.

#### Secondary outcomes at 24 months

- Proportion of eyes with central macular subfield thickness <250 µm in the overall study and in each randomisation arm.
- Number of intravitreal anti-VEGF injections in each randomisation arm.
- Number of laser sessions needed for DMO in each randomisation arm.
- BCVA letter score gains in the overall study and in each randomisation arm.
- Rates of adverse events such as cataract, raised intraocular pressure, endophthalmitis, myopathy, elevated serum creatinine and liver enzymes, venous thrombotic events and cardiovascular events in each randomisation arm.
- Progression (two-step and three-step progression) of DR lesions in each randomisation arm.
- Change in quality of life measures (assessed using National Eye Institute Visual Function Questionnaire 25 (NEI-VFQ 25) and EuroQol-five dimension (EQ-5D) questionnaire) in each randomisation arm from baseline to 24 months.

#### Measurement of outcomes and other variables

The main activities at each mandated visit are summarised in table 1. Study visits will occur at recruitment (baseline) and 3, 6, 12, 18 and 24 months (six visits in total). Centres will be encouraged to review patients at least every 2 months, but apart from the key mandated visits, review frequency will be decided by treating investigators according to their usual practice.

**Table 1:** Participant timeline and schedule of visit in the fenofibrate for the treatment of diabetic macular oedema study

Procedures	Screening/baseline	Month 1 (week 4)	Month 3 (week 12)	Month 6 (week 24)	Month 12 (week 48)	Month 18 (week 72)	Month 24 (week 96)
Visit number	1	Phone call	2	3	4	5	6
Visit/call window (days)	1	±7	±30	±30	±30	±30	±30
Informed consent	**x**						
Assign patient number	**x**						
Randomisation	**x**						
Demographics and medical history questionnaire	**x**						
EuroQol-five dimension questionnaire	**x**				**x**		**x**
National Eye Institute Visual Function Questionnaire-25 questionnaire	**x**				**x**		**x**
Pregnancy test	**x**						
Blood samples	**x**			**x**	**x**		**x**
Vital signs	**x**		**x**	**x**	**x**	**x**	**x**
Best-corrected visual acuity	**x**		**x**	**x**	**x**	**x**	**x**
Intraocular pressure	**x**		**x**	**x**	**x**	**x**	**x**
Spectral domain-OCT imaging	**x**		**x**	**x**	**x**	**x**	**x**
Fundus photographs	**x**			**x**	**x**		**x**
Ophthalmic examination	**x**		**x**	**x**	**x**	**x**	**x**
Fluorescein angiography/OCT angiography	**x**				**x**		**x**
Concomitant medications	**x**	**x**	**x**	**x**	**x**	**x**	**x**
Adverse events		**x**	**x**	**x**	**x**	**x**	**x**

OCToptical coherence tomography

At the recruitment visit, detailed information on patient demographics and medical history will be obtained by interview and medical record review. This includes age, gender, ethnicity, ocular history (previous treatment for DMO or DR, previous surgery, current eye drops, refractive error, cataract and glaucoma), medical history (diabetes duration, diabetic control, renal impairment and cardiovascular disease) and medication history. At the baseline and 6-, 12- and 24-month visits, non-fasting blood samples will be taken for measurement of full blood counts, general chemistry, plasma glucose, glycated haemoglobin (HbA1c), cholesterol and triglyceride levels and liver and renal function. At baseline and 12- and 24-month visits, the NEI-VFQ 25 and EQ-5D questionnaires will be administered.^23 24^ These are reliable and valid instruments to capture visual function and quality of life in people with DMO.^25 26^

At each key mandated visit, participants will undergo a clinical eye and vital sign examination. BCVA will be measured using standard trial quality ETDRS visual acuity charts.^27^ The measurements will be obtained by a certified technician masked to treatment assignment. Slit lamp examination of anterior and posterior segments will be performed, and details of intraocular pressure, cataract, neovascularisation, vitreous haemorrhage, clinical severity of DR and other relevant pathology will be documented. Posterior segment colour and red-free fundus photographs of ETDRS fields 1–7 will be taken at each key visit using a high-resolution Canon CR-DGI camera (Canon, Tokyo, Japan) following maximal pupil dilation with 1% tropicamide. All patients will undergo SD-OCT scanning using the Spectralis HRA+OCT machine with viewing module V.5.1.2.0 (Heidelberg Engineering, Heidelberg, Germany). Scan protocols will include a dense horizontal linear scan centred on the fovea, and the HEYEX software interface (V.1.6.2.0; Heidelberg Engineering) will be used to correlate position markers on near-infrared to facilitate registration and longitudinal scanning of the same area at each subsequent visit. Photographs will be graded for DR using the ETDRS adaptation of the modified Airlie House classification.^27^

#### Rescue treatment and adverse outcomes of specific interest

Rescue treatment will be permitted with intravitreal triamcinolone or dexamethasone implant or surgical vitrectomy following three intravitreal anti-VEGF injections if, in the opinion of the treating investigator, there has been no significant clinical improvement in either BCVA or central macular subfield thickness.

Fenofibrates are a well-tolerated drug with low rates of adverse events.^28^ The main adverse events of special interest are as follows.

- Myopathy: there are some concerns that myositis and rhabdomyolysis may occur at increased rates when fenofibrate is taken together with statin therapy. Participants will be questioned regarding muscle pain, tenderness and weakness at each visit, and if present, will have serum creatine kinase levels measured. As per Therapeutic Goods Administration guidelines,^29^ if levels are >10× normal limits, treatment will be discontinued and randomisation unmasked.
- Elevated liver transaminases and creatinine: these will be routinely monitored at the 6-, 12- and 24-month visits. Following Therapeutic Goods Administration guidelines,^29^ treatment will be discontinued if liver transaminases are raised 3× above normal limits or serum creatinine is raised 1.5× above normal limits.

All adverse events, regardless of relatedness to the study drug, will be recorded with the severity grade, suspected relationship to the study drug, duration, whether it constitutes a serious adverse event and site of adverse event.

### Economic evaluation

Medicare Benefits Scheme costs will be captured prospectively during the study through linked data. Utility scores will be calculated based on the EQ-5D questionnaire applied to patients at baseline and 12- and 24-month visits. The main outcome measure is the incremental cost-effectiveness ratio (ICER), expressed as the additional cost per quality-adjusted life year (QALY) gain by fenofibrate over placebo. The results will be plotted on a cost-effectiveness plane. To reflect the variation in mean costs and outcomes, bootstrapping will be used to estimate a distribution around these variables and to calculate the CIs around the ICER. One-way sensitivity analysis will be conducted around key variables, and a probabilistic sensitivity analysis will be conducted to estimate the joint uncertainty in all parameters. Trial-based data, however, cannot capture costs and outcomes beyond the trial. To address this, a modelled economic evaluation will enable quality of life and survival to be examined and allow ICERs to be calculated in terms of cost per QALY gained over 15 years in a hypothetical cohort of patients with DMO. Healthcare costs will include the costs of treatment and ongoing monitoring from the Medicare Benefits Schedule and Pharmaceutical Benefits Scheme).

### Sample size considerations

Assuming a 15% dropout rate over 2 years and 10% non-compliance with fenofibrate therapy, recruitment of 204 participants (102 fenofibrate+102 placebo) with DMO will provide 80% power at a 5% significance level to detect our primary outcome of a 20% (76 µm) reduction in mean macular thickness overall for fenofibrate versus placebo, assuming mean baseline central macular subfield thickness of 380 µm with SD 110 µm.

### Data collection and analysis plan

The primary outcome is the 24-month reduction in mean central macular subfield thickness in all patients on fenofibrate compared with all controls. Analyses will subsequently be performed in prespecified stratified subgroups for DMO requiring clinical observation and DMO requiring treatment, and further among anti-VEGF-treated and laser-treated DMO. Intention-to-treat analyses will use analysis of covariance (ANCOVA) adjusting for baseline values and mixed models for repeated measures, which accommodate data with missingness at random. Survival analyses will be used to estimate and compare the time to achieve the secondary outcome of central macular subfield thickness <250 µm and the proportions who achieve that reduction over the 24-month period. Multiple regression analyses adjusting for age, HbA1c, number of injections and other covariates will also be performed.

Baseline characteristics of patients randomised to fenofibrate and placebo will be tabulated and compared using χ^2^ tests for categorical variables and t-tests for continuous variables. All main outcome analyses will be by intention-to-treat. Per-protocol exploratory and propensity analyses may also be conducted. The mean gain in ETDRS visual acuity letter scores in patients on fenofibrate will be compared with placebo in all patients, followed by analyses in DMO requiring treatment and DMO requiring observation groups. Analyses will be by intention-to-treat and will use ANCOVA and mixed models for repeat measures. Statistical analysis will be performed using SAS V.9.4 (SAS Institute, North Carolina, USA).

### Data management

Designated study personnel will enter the clinical data into the case report forms (CRF). Following sufficient training, these personnel will transfer the data onto electronic CRFs (eCRFs) using fully validated software. The study personnel must certify that the data entered into the eCRF are complete and accurate. All paper copies of data information will be physically stored in locked cabinets. Information stored on computer files will have a password that is known only to the key study personnel. All patients enrolled in the study will be given a unique patient ID number, which will be used to de-identify them following the collection of relevant research parameters. Data will be stored for 20 years, as required by law for clinical trials, with only the key study personnel having access.

### Ethics and dissemination

Ethics approval has been granted by the University of Sydney Human Ethics Committee (HREC-2019/892), and the trial has been registered with the Australia New Zealand Clinical Trials Registry (ACTRN12618000592246). The study adheres to the principles of the Helsinki Declaration and the NHMRC National Statement on Ethical Conduct in Human Research. Trial results will be disseminated to the public in de-identified form through publications in peer-reviewed journals.

## supplementary material

10.1136/bmjopen-2024-089518 online supplemental file 1

## References

[1] Dirani M (2013). Out of sight: a report into diabetic eye disease in australia. https://baker.edu.au/-/media/documents/impact/outofsightreport.pdf.

[2] Yuen YS, Gilhotra JS, Dalton M (2023). Diabetic Macular Oedema Guidelines: An Australian Perspective. J Ophthalmol.

[3] Cooper OAE, Taylor DJ, Crabb DP (2020). Psychological, social and everyday visual impact of diabetic macular oedema and diabetic retinopathy: a systematic review. Diabet Med.

[4] Lee CMY, Colagiuri R, Magliano DJ (2013). The cost of diabetes in adults in Australia. Diabetes Res Clin Pract.

[5] Bahrami B, Zhu M, Hong T (2016). Diabetic macular oedema: pathophysiology, management challenges and treatment resistance. Diabetologia.

[6] Wong TY, Simó R, Mitchell P (2012). Fenofibrate - a potential systemic treatment for diabetic retinopathy?. Am J Ophthalmol.

[7] Noonan JE, Jenkins AJ, Ma J-X (2013). An update on the molecular actions of fenofibrate and its clinical effects on diabetic retinopathy and other microvascular end points in patients with diabetes. Diabetes.

[8] Gao L, Zhang Y, Wang X (2021). Association of apolipoproteins A1 and B with type 2 diabetes and fasting blood glucose: a cross-sectional study. BMC Endocr Disord.

[9] Giurdanella G, Lupo G, Gennuso F (2020). Activation of the VEGF-A/ERK/PLA2 Axis Mediates Early Retinal Endothelial Cell Damage Induced by High Glucose: New Insight from an In Vitro Model of Diabetic Retinopathy. Int J Mol Sci.

[10] Kim J, Ahn J-H, Kim J-H (2007). Fenofibrate regulates retinal endothelial cell survival through the AMPK signal transduction pathway. Exp Eye Res.

[11] Garcia-Ramírez M, Hernández C, Palomer X (2016). Fenofibrate prevents the disruption of the outer blood retinal barrier through downregulation of NF-κB activity. Acta Diabetol.

[12] Cacicedo JM, Benjachareonwong S, Chou E (2011). Activation of AMP-activated protein kinase prevents lipotoxicity in retinal pericytes. Invest Ophthalmol Vis Sci.

[13] Meissner M, Stein M, Urbich C (2004). PPARalpha activators inhibit vascular endothelial growth factor receptor-2 expression by repressing Sp1-dependent DNA binding and transactivation. Circ Res.

[14] Chen Y, Hu Y, Lin M (2013). Therapeutic effects of PPARα agonists on diabetic retinopathy in type 1 diabetes models. Diabetes.

[15] Keech AC, Mitchell P, Summanen PA (2007). Effect of fenofibrate on the need for laser treatment for diabetic retinopathy (FIELD study): a randomised controlled trial. Lancet.

[16] Chew EY, Davis MD, Danis RP (2014). The effects of medical management on the progression of diabetic retinopathy in persons with type 2 diabetes: the Action to Control Cardiovascular Risk in Diabetes (ACCORD) Eye Study. Ophthalmology.

[17] Chew EY, ACCORD Study Group, ACCORD Eye Study Group (2010). Effects of medical therapies on retinopathy progression in type 2 diabetes. N Engl J Med.

[18] Massin P, Peto T, Ansquer J-C (2014). Effects of fenofibric acid on diabetic macular edema: the MacuFen study. Ophthalmic Epidemiol.

[19] Srinivasan S, Hande P, Shetty J (2018). Efficiency of fenofibrate in facilitating the reduction of central macular thickness in diabetic macular edema. Indian J Ophthalmol.

[20] Wells JA, Glassman AR, Diabetic Retinopathy Clinical Research Network (2015). Aflibercept, bevacizumab, or ranibizumab for diabetic macular edema. N Engl J Med.

[21] Mitchell P, Bandello F, Schmidt-Erfurth U (2011). The RESTORE study: ranibizumab monotherapy or combined with laser versus laser monotherapy for diabetic macular edema. Ophthalmology.

[22] Comyn O, Heng LZ, Ikeji F (2012). Repeatability of Spectralis OCT measurements of macular thickness and volume in diabetic macular edema. Invest Ophthalmol Vis Sci.

[23] Mangione CM, Lee PP, Gutierrez PR (2001). Development of the 25-item National Eye Institute Visual Function Questionnaire. Arch Ophthalmol.

[24] Devlin NJ, Brooks R (2017). EQ-5D and the EuroQol Group: Past, Present and Future. Appl Health Econ Health Policy.

[25] Lloyd AJ, Loftus J, Turner M (2013). Psychometric validation of the Visual Function Questionnaire-25 in patients with diabetic macular edema. Health Qual Life Outcomes.

[26] McGuinness MB, Ayton LN, Schofield D (2024). EQ-5D-5L health utility scores in Australian adults with inherited retinal diseases: A cross-sectional survey. Acta Ophthalmol.

[27] (1991). Grading Diabetic Retinopathy from Stereoscopic Color Fundus Photographs—An Extension of the Modified Airlie House Classification. ETDRS report number 10. Early Treatment Diabetic Retinopathy Study Research Group. Ophthalmology.

[28] Guo J, Meng F, Ma N (2012). Meta-analysis of safety of the coadministration of statin with fenofibrate in patients with combined hyperlipidemia. Am J Cardiol.

[29] Therapeutic Goods Administration (2006). LIPIDIL fenofibrate 145 mg tablet blister pack. https://www.tga.gov.au/resources/artg/118634.

