# Supplementary material for: Randomised, multicentre, placebo-controlled trial of fenofibrate for treatment of diabetic macular oedema with economic evaluation (FORTE study): study protocol for a randomised control trial
Source: BMJ Open. 2024 Dec 20;14(12):e089518. doi: 10.1136/bmjopen-2024-089518 (PMC11683925; doi:10.1136/bmjopen-2024-089518)
Supplement: online supplemental file 1 [file bmjopen-14-12-s001.pdf]

THE UNIVERSITY OF SYDNEY

Discipline of Opthalmology
Faculty of Medicine

ABN 15 211 513 464

ASSOCIATE PROFESSOR GERALD LIEW
Clinical Associate Professor and Ophthalmologist
PROFESSOR PAUL MITCHELL
Professor and Ophthalmologist

Room 0.3.14
The Westmead Institute
For Medical Research
The University of Sydney
NSW 2006 AUSTRALIA
Telephone: +61 2 8627 3365
Facsimile: +61 2 8627 3099
Email: gerald.liew@sydney.edu.au
Web: http://www.sydney.edu.au/

A Randomised Multi-centre Placebo Controlled Trial of Fenofibrate for Treatment of Diabetic Macular Oedema with Economic Evaluation (FORTE Study)

PARTICIPANT CONSENT FORM

I, ... [PRINT NAME], agree to take part in this research study.

In giving my consent I state that:

- ✓ I understand the purpose of the study, what I will be asked to do, and any risks/benefits involved.
- ✓ I have read the Participant Information Statement and have been able to discuss my involvement in the study with the researchers if I wished to do so.
- ✓ The researchers have answered any questions that I had about the study and I am happy with the answers.
- ✓ I understand that being in this study is completely voluntary and I do not have to take part. My decision whether to be in the study will not affect my relationship with the researchers, anyone else at the University of Sydney, or my treating Ophthalmologist, now or in the future.
- ✓ I understand that I can withdraw from the study at any time.
- ✓ I understand that I may stop the interviewer-administered questionnaire at any time if I do not wish to continue. I understand that I must indicate if I wish for any of my records to be erased so that the information I have provided will not be included in the study. I also understand that I may refuse to answer any questions I don't wish to answer.

✓ I understand that personal information about me that is collected over the course of this project will be stored securely and will only be used for purposes that I have agreed to. I understand that information about me will only be told to others with my permission, except as required by law.

✓ I understand that the results of this study may be published, and that publications will not contain my name or any identifiable information about me.

I consent to:

• **Having my blood taken**	YES	☐	NO	☐
• **Having my blood used for specific genetic testing**	YES	☐	NO	☐
• **Being informed of the results of the genotyping**	YES	☐	NO	☐
• **Receiving feedback about my personal results**	YES	☐	NO	☐

Would you like to receive feedback about the overall results of this study?

YES ☐ **NO** ☐

If you answered **YES**, please indicate your preferred form of feedback and address:

☐ Postal: ____

____

☐ Email: ____

....
Signature

....
PRINT name

....
Date
