# Supplementary material for: Randomised, multicentre, placebo-controlled trial of fenofibrate for treatment of diabetic macular oedema with economic evaluation (FORTE study): study protocol for a randomised control trial
Source: BMJ Open. 2024 Dec 20;14(12):e089518. doi: 10.1136/bmjopen-2024-089518 (PMC11683925; doi:10.1136/bmjopen-2024-089518)
Supplement: Supplementary file 2 [file bmjopen-2024-089518.reviewer_comments.pdf]

PEER REVIEW HISTORY

BMJ Open publishes all reviews undertaken for accepted manuscripts. Reviewers are asked to complete a checklist review form and are provided with free text boxes to elaborate on their assessment. These free text comments are reproduced below.

ARTICLE DETAILS

Title (Provisional)

A Randomised, Multi-centre, Placebo Controlled Trial of Fenofibrate for Treatment of Diabetic Macular Edema with Economic Evaluation (FORTÉ Study): Study Protocol for a Randomised Control Trial.

Authors

Nguyen, Helen; Kha, Richard; Gopinath, Bamini; Mitchell, Paul; Liew, Gerald

---

VERSION 1 - REVIEW

Reviewer 1

Name Lai, kunbei

Affiliation Sun Yat-Sen University Zhongshan Ophthalmic Center State Key Laboratory of Ophthalmology

Date 18-Aug-2024

COI None

It is an interesting article focused on Fenofibrate for the treatment of diabetic macular edema, which may provide a new approach for the combination therapy of DME. However, some issues need to be revised and improved by the author before publication:

1. Discussion: The possible mechanism of fenofibrate in the treatment of DME should be discussed in more details.

2. Detailed baseline data: such as severity of DR, duration of DR, blood lipid levels.

3. The follow-up timepoint of Month 1, Month2 is also important for a randomised control trial, please explain why is missing.

Reviewer 2

Name Delhiwala, Kushal

Affiliation Netralaya Superspeciality Eye Hospital, Vitreo retina and uvea

Date 25-Aug-2024

**COI**	**none**

The study protocol is looking good to provide some new data pertaining to the effect of fenofibrate in managing diabetic macular edema, which so far has largely remained unanswered.

**Reviewer**	**3**
**Name**	**Sadiq, Salman**
**Affiliation**	**Royal Victoria Infirmary, Ophthalmology**
**Date**	**25-Aug-2024**
**COI**	**None to declare**

Dear Authors

Thank you for your effort in developing this trial, which promises to significantly contribute to the evidence supporting the use of Fenofibrate in patients with established DME. We are aware of the RCT from Scotland that explored the effects of Fenofibrate in a screening diabetic population with non-referrable DR. However, your trial's focus on its role in established DME is particularly compelling, and we look forward to seeing the results.

I have a few minor suggestions for your consideration:

1. Spelling Correction (Line 44):*Please correct the word "Scam" to "Scan."

2. Citation Addition: It may be beneficial to cite the following paper in your protocol, as it shares some similarities in design and utilizes a slightly higher dose of Fenofibrate:

Srinivasan, Srilakshmi; Hande, Prathibha; Shetty, Jyoti; Murali, Sindhu. "Efficiency of fenofibrate in facilitating the reduction of central macular thickness in diabetic macular edema." Indian Journal of Ophthalmology, 66(1):98-105, January 2018. DOI: 10.4103/ijo.IJO_566_17.

Thank you once again for your contributions to this area of study.

VERSION 1 - AUTHOR RESPONSE

Reviewer 1 Comments

Comment 1. Discussion: The possible mechanism of fenofibrate in the treatment of DME should be discussed in more details.

Response 1. Thank you for the comment. The mechanism of fenofibrate in the treatment of diabetic eye disease has been further elaborated in the manuscript.

Page 5, Lines 92-113. "Fenofibrate is a peroxisome proliferator activated-receptor alpha (PPAR-α) agonist which shows promise for the treatment for DME. It is an orally delivered fibric acid which has been used to treat dyslipidaemia for decades. It has an excellent safety profile, is well tolerated and inexpensive.[6] After absorption, fenofibrate activates PPAR-α which is a transcription factor responsible for the expression of genes regulating lipid metabolism.[7] Activation of PPAR-α by fenofibrate upregulates the synthesis of proteins resulting in reduced low-density lipoproteins and triglyceride levels and increased high-density lipoprotein levels.[7]

The mechanism by which fenofibrate inhibits progression of diabetic eye disease remains unknown and is thought to be more complex than the serum lipid-lowering effect. Fenofibrate upregulates apolipoprotein A1, which may have a role in reducing progression of DR by protecting the retina from oxidative stress and lipotoxicity.[8] Fenofibrate also lowers lipoprotein-associated phospholipase A2 which has been shown to decrease production of pro-angiogenic prostaglandins and retinal neovascularisation.[9] In addition to these lipid-modulating effects, several other non-lipid mechanisms have been proposed to explain the protective effect of fenofibrate in DR. These include decreased endothelial cell permeability, interleukin-1 beta-mediated retinal pigment epithelium (RPE) disruption and nuclear factor kappa B (NF-κB)-mediated inflammatory cytokine expression, all of which contribute to blood retinal breakdown in DME.[10, 11] Fenofibrate also downregulates stress-related signalling and apoptosis of endothelial and pericyte cells through adenosine monophosphate-activated protein kinase (AMPK)-dependent pathways to preserve the outer blood retinal barrier.[10, 12] Moreover, fenofibrate may have a potential role in inhibiting angiogenesis by downregulating vascular endothelial growth factor (VEGF) in RPE cells.[13, 14]"

Comment 2. Detailed baseline data: such as severity of DR, duration of DR, blood lipid levels.

Response 2. Thank you for the comment. The study is ongoing, and data collection is continuing. As such, the database has not been finalised or locked. We would prefer to present the baseline data in a later publication, once follow-up of all participants is completed and the database is locked. This would ensure that only one validated, data cleaned, and verified final dataset will be used for all analyses. As this is a multi-centre study, the baseline data from different centres has not been fully validated, with source document verification and data reconciliation ongoing.

Nonetheless, if the reviewers believe this baseline data to be necessary, we will be happy to provide this based on the current dataset, with a proviso that the final, locked dataset analyses may differ.

Comment 3. The follow-up timepoint of Month 1, Month2 is also important for a randomised control trial, please explain why is missing.

Response 3. Thank you for the comment. We agree, and in the initial protocol, we conducted month 1 mandated study visits. However soon after commencement of the study, the Covid-19 pandemic occurred, which resulted in multiple lock downs over a period of 2 years. The protocol was then modified to minimise face to face participant visits, both because the lock downs prevented in person visits, and to minimise risk of spreading infection. The month 1 visit was converted to a phone call to ensure any serious adverse events were detected and address any participant queries. If there were any concerns, an in person flexible visit was arranged. The loss of month 1 imaging and review was not believed to affect the final results

as the beneficial effects of oral fenofibrate become apparent only after 6 months of continuous use, as shown in the FIELD and other prior studies.

Reviewer 3 Comments

Thank you for your effort in developing this trial, which promises to significantly contribute to the evidence supporting the use of Fenofibrate in patients with established DME. We are aware of the RCT from Scotland that explored the effects of Fenofibrate in a screening diabetic population with non-referrable DR. However, your trial's focus on its role in established DME is particularly compelling, and we look forward to seeing the results.

I have a few minor suggestions for your consideration:

Comment 1. Spelling Correction (Line 44): Please correct the word "Scam" to "Scan."

Response 1. Thank you for the correction! The word has been corrected to 'scan.'

Page 11, Line 252. "OCT scan protocols will include a dense horizontal linear scan centred on the fovea…"
Comment 2. Citation Addition: It may be beneficial to cite the following paper in your protocol, as it shares some similarities in design and utilizes a slightly higher dose of Fenofibrate:
Srinivasan, Srilakshmi; Hande, Prathibha; Shetty, Jyoti; Murali, Sindhu. "Efficiency of fenofibrate in facilitating the reduction of central macular thickness in diabetic macular edema." Indian Journal of Ophthalmology, 66(1):98-105, January 2018. DOI: 10.4103/ijo.IJO_566_17.

Response 2. Thank you for this suggestion and bringing the study to our attention, we now cite this study in the Discussion section of the manuscript.

Page 6, Lines 134-138. "Another study by Srinivasan et al. in 2018 evaluated the effect of oral fenofibrate on macular thickness and visual function in 50 participants with DME.[16] This study found a statistically significant decrease in macular thickness after 6 months but no improvement in visual function.[16] However, the study was limited by its small sample size and short follow-up duration of 6 months."

VERSION 2 - REVIEW

Reviewer **1**

Name **Lai, kunbei**

Affiliation **Sun Yat-Sen University Zhongshan Ophthalmic Center State Key Laboratory of Ophthalmology**

Date **15-Nov-2024**

COI

Looks good now.
